# Single-Cell Spatial MIST for Versatile, Scalable Detection of Protein Markers

**DOI:** 10.3390/bios13090852

**Published:** 2023-08-27

**Authors:** Arafat Meah, Vadanasundari Vedarethinam, Robert Bronstein, Nehaben Gujarati, Tanya Jain, Sandeep K. Mallipattu, Yueming Li, Jun Wang

**Affiliations:** 1 Multiplex Biotechnology Laboratory, Department of Biomedical Engineering, State University of New York at Stony Brook, Stony Brook, NY 11794, USA; 2 Division of Nephrology and Hypertension, Department of Medicine, Stony Brook School of Medicine, Stony Brook, NY 11794, USA; 3 Chemical Biology Program, Memorial Sloan Kettering Cancer Center, New York, NY 10065, USA; 4 Programs of Neurosciences, Weill Graduate School of Medical Sciences of Cornell University, New York, NY 10065, USA; 5 Renal Section, Northport VA Medical Center, Northport, NY 11768, USA; 6 Programs of Pharmacology, Weill Graduate School of Medical Sciences of Cornell University, New York, NY 10021, USA

**Keywords:** multiplex biosensor, spatial proteomics, single-cell analysis, protein markers, MIST array

## Abstract

High-multiplex detection of protein biomarkers across tissue regions has been an attractive spatial biology approach due to significant advantages over traditional immunohistochemistry (IHC) methods. Different from most methods, spatial multiplex in situ tagging (MIST) transfers the spatial protein expression information to an ultrahigh-density, large-scale MIST array. This technique has been optimized to reach single-cell resolution by adoption of smaller array units and 30% 8-arm PEG polymer as transfer medium. Tissue cell nuclei stained with lamin B have been clearly visualized on the MIST arrays and are colocalized with detection of nine mouse brain markers. Pseudocells defined at 10 μm in size have been used to fully profile tissue regions including cells and the intercellular space. We showcased the versatility of our technology by successfully detecting 20 marker proteins in kidney samples with the addition of five minutes atop the duration of standard immunohistochemistry protocols. Spatial MIST is amenable to iterative staining and detection on the same tissue samples. When 25 proteins were co-detected on 1 mouse brain section for each round and 5 rounds were executed, an ultrahigh multiplexity of 125 proteins was obtained for each pseudocell. With its unique abilities, this single-cell spatial MIST technology has the potential to become an important method in advanced diagnosis of complex diseases.

## 1. Introduction

Advanced single-cell tools have revolutionized the biomedical sciences, as they can survey a whole spectrum of molecules across a heterogenous cellular makeup. Most of these tools are based on either RNA/DNA sensing or protein sensing with a multiplexity even reaching the omics scale. The employed sequencing method essentially acts as an array-based biosensor, designed to elucidate genome-wide gene expression across various cells [1,2]. However, proteins that carry out the ‘real work’ for cells have not yet been detected at a similar scale, especially for tissue cells. Proteins more accurately reflect cell functions and are biomarkers for disease diagnosis, cell type classification and therapeutic targets [3]. The individual cell’s proteome provides a functional read-out of cell identity, drug targets, clinical biomarkers, signaling networks, transcription factors, cell proliferation, cell cycle status, metabolism and injury [3,4]. In disease pathology and signal pathway analyses, single-cell functional proteomics is irreplaceable because of the poor correlation between gene expression and protein expression, especially for low expression proteins such as signaling proteins [5,6,7]. Furthermore, protein-level diagnosis offers more direct information regarding disease pathogenesis and potential drug targets [4].

In light of the high impact generated by single-cell transcriptomics, a counterpart single-cell proteomics tool is in high demand to generate the much-needed pathophysiological information and decipher disease mechanisms [8]. The current single-cell protein assay technologies represented by mass cytometry (CyTOF and Imaging Mass Cytometry) and multiparameter flow cytometry can only analyze dozens of proteins, which is far from the genome scale. Similar tools are also either time consuming (e.g., CycIF and CODEX) [9,10] or require special instruments (e.g., mass analyzer for MIBI) [11] that are not available/affordable for common laboratories. The instrument of mass spectrometry has been improved recently to possess the capability of measuring more than 1000 protein types in single mammalian cells by SCoPE-MS and nanoPOTS [8,12,13]. However, the proteins that are readily measurable typically include the most abundant ones, such as enzymes and cytoskeletal proteins. Conversely, functional proteins—such as surface receptors, transcription factors and signaling proteins—often exist in low quantities, rendering many of them undetectable with these methods. In addition, single-cell mass spectrometry lacks spatial context for the cells due to the aggregate sample treatment process. Other high-content mass spectrometry only achieves the spatial resolution of about 50–100 μm, which is much larger than the size of a typical cell [14].

Herein, we introduce a single-cell spatial multiplex in situ tagging (MIST) technology that can reach a single-cell resolution and detect over 100 protein markers with high sensitivity. This technology is built upon our spatial MIST platform but with substantial improvements. The single-cell spatial MIST technology accurately prints the shape of nuclei on the MIST array. Simultaneously, it colocalizes detection signals from various proteins, streamlining the profiling of protein expression in individual cells from tissue samples. This technology is also highly versatile as it is applicable to multiple tissue sources including brain and kidney sections. We demonstrated the ability of single-cell spatial MIST in detection of 125 protein markers through an iterative staining technique. We non-discriminately analyzed protein marker profiles of pseudocells to ensure a comprehensive investigation of all tissue regions, including areas beyond cells, such as the intercellular spaces. Future biological studies leveraging our technology are anticipated to yield unique mechanistic insights on a functional proteomic scale.

## 2. Materials and Methods

### 2.1. Microbead Array Preparation

Polystyrene beads with carboxyl groups (4% *w*/*v* 1.4 μm, Life Sciences Technologies, Hewlett Harbor, NY, USA) were first conjugated with DNA oligonucleotides that contain 2 sections. The 9 oligo DNAs (30–40 bases for each) were custom-designed and purchased from Integrated DNA Technologies. Each oligonucleotide sequence contains sequence 35 (Table S1), which serves as a cell marker, along with another oligo sequence for the detection of 1 of 9 proteins. The exact oligonucleotide sequences used for all experiments can be found in Table S1. Polystyrene beads with amine groups (2 μm; Life Science Technologies, Hewlett Harbor, NY, USA) were conjugated with one-section oligonucleotides.

For the preparation of 100 μL of carboxyl latex beads, 0.5 mg/mL of 1-ethyl-3-(3-dimethylaminopropyl) carbodiimide hydrochloride (EDC, Thermo Fisher, Fair Lawn, NJ, USA) and 0.5 mg/mL of Sulfo-NHS (N-hydroxysulfosuccinimide, Thermo Fisher, Fair Lawn, NJ, USA) were first mixed in pH 4.7 MES buffer and incubated for 1 h to form an amine-reactive NHS group on the bead surface. For amine-bearing beads, microbeads were treated with 10 mM bis(sulfosuccinimidyl)suberate (BS3; Pierce, Thermo Fisher, Fair Lawn, NJ, USA) crosslinker solution for 20 min before centrifugation and washing thoroughly. Next, the beads were incubated with a mixture of 0.05% of Poly-L-lysine solution (PLL; Ted Pella, Redding, CA, USA) and allowed to incubate for 2 h in an orbital shaker. After washing of the beads, 5 mM Azido-PEG4-NHS (Click Chemistry, Scottsdale, AZ, USA) were added to the microbeads and were left to incubate on an orbital shaker for 4 h. Concurrently, 150 μM ssDNA was reacted with 50 mM DBCO-NHS (Click Chemistry, Scottsdale, AZ, USA) at pH 8.5 for 4 h at room temperature (RT). After both reactions were finished, microbeads were washed with PBS while the modified oligo DNAs were purified through a 7 k MWCO zeba spin column (Thermo Fisher, Fair Lawn, NJ, USA). The beads were then resuspended in the DNA solution and allowed to react overnight in an orbital shaker. Finally, once the reaction was complete, the beads were washed with Milli Q water and resuspended to their original concentration. The microbeads carrying different oligonucleotides were then mixed in equal portions and attached to an adhesive tape (VWR, Radnor, PA, USA) on a glass slide. The surface area of the array could be adjusted by either increasing or decreasing the amount of bead added to the tape. To ensure the quality of the arrays, each of them has been examined under a microscope to ensure monolayer and compact packing (Figure S1).

The sensitivity of the MIST array was characterized by Cy5-labeled complementary DNAs (cDNAs) in tris buffer with 0.05% of Tween 20 (TBST). After incubation for 1 h, the arrays were washed with TBST five times. A calibration curve was made for each DNA using different Cy5-cDNA at concentrations ranging from 10 pM to 100 nM. A logistic function was utilized to model the relation between the varying concentrations and the fluorescence intensities of the microbeads. The limit of detection (LOD) was determined by summing the background noise with three times its standard deviation based on the fitting curve.

### 2.2. Preparation and Purification of UV-Cleavable, Biotinylated DNA-Antibody Conjugates

The conjugation of biotin-tagged oligo DNAs with antibodies was executed through a DBCO-azido click chemistry reaction [15]. All antibodies that were used are listed in Table S1. The antibodies were initially concentrated to 1 mg/mL by utilizing 10 K MWCO Amicon centrifuge filters (Thermo Fisher, Fair Lawn, NJ, USA). Subsequently, 50 μL of antibodies underwent modification using an azido-NHS ester with a UV cleavable linker (Click Chemistry Tools, Scottsdale, AZ, USA) at a 1:15 molar ratio for 2 h. Concurrently, the amine-terminated, biotin-tagged DNA solutions with a concentration of 200 μM were pH-balanced to 8.0 and underwent conjugation with a DBCO-NHS ester with a UV cleavable linker (Click Chemistry Tools, Scottsdale, AZ, USA) at a 1:20 molar ratio for 2 h. DNAs with a hybridized chain reaction (HCR) initiator sequence were first snap cooled before modification. The modified antibodies and oligo DNAs were then purified by 7 K MWCO Zeba spin desalting columns, mixed and incubated overnight at ambient temperature. These conjugates were purified by a FPLC device (AKTA model, manufactured by Bio-Rad, Hercules, CA, USA). The harvested compounds were concentrated to a range of 0.3–0.5 mg/mL using a Nanodrop spectrophotometer (Thermo Fisher, Fair Lawn, NJ, USA) and were refrigerated at 4 °C for future application.

### 2.3. Spatial MIST Detection and Characterization

Perfusion fixed brain tissue sections from C57BL/6J male mice were acquired from the Memorial Sloan Kettering Center. To acquire kidney tissue from wildtype and diabetic mice, 8-week-old db/+ and db/db mice (FVB/N background) (Stony Brook University) were initially perfused with PBS and subsequently formalin-fixed prior to sectioning. Tissue sections were preheated in a 70 °C oven for 30 min before deparaffinization. Deparaffinization of formalin-fixed, paraffin-embedded (FFPE) tissue samples involved two 5-min xylene (Thermo Fisher, Fair Lawn, NJ, USA) washes to dissolve the paraffin. The xylene was then removed by 3-min immersions in 1:1 xylene: ethanol then 100% ethanol (Thermo Fisher, Fair Lawn, NJ, USA). Tissues were rehydrated through a series of 3-min washes in 95%, 70% and finally 50% ethanol, respectively. A 5-min rinse in distilled water completed the deparaffinization process. Subsequently, tissue sections were then submerged in pH 8 Tris-EDTA at 95 °C for 10 min, washed with cool PBS and permeabilized with 0.3% Triton X-100 for 10 min. After permeabilization, tissue sections were incubated in a blocking solution composed of 5% goat serum, 100 μg/mL fragmented salmon sperm DNA (Thermo Fisher, Fair Lawn, NJ, USA) and 0.05% Tween 20 in PBS for 1 h. Next, the brain slices were incubated with 100-fold diluted conjugates, and incubated for 3 h in a dark room at RT. Simultaneously, a MIST array bearing microbeads coated with cDNA was blocked using a TBST buffer for 10 min. Following three washes, various concentrations of 8-Arm PEG-OH (MW 40 k, Creative PEGWorks, Durham, NC, USA) were added to both the tissue slice and the MIST array. After 10 min of incubation, the MIST array and tissue slice were mated together and firmly secured with a magnetic clamp. The complete assembly was exposed to UV light (365 nm, sourced from Thorlabs CS2010 UV Curing LED System, Newton, NJ, USA) for 5 min before careful detachment in a PBS buffer. The MIST array and tissue were rigorously rinsed with PBS buffer. The MIST array underwent either hybridization chain reaction (HCR) amplification (carboxyl latex) or protein amplification (amine latex) in the next step. For HCR-based signal detection, HCR hairpins (B1-H1-488 3 μM, B2-H2-488 3 μM; Molecular Instruments) were snap cooled by being heated at 95 °C for 90 s then allowed to cool for 30 min at RT. During this time, HCR amplifier buffer (Molecular Instruments, Los Angeles, CA, USA) was added to the MIST array for 30 min. Each hairpin was diluted separately to 60 nM with HCR amplifier buffer. Graphene Oxide (Thermo Fisher, Fair Lawn, NJ, USA) was added in each initiator at a concentration of 20 μg/mL. The addition of Graphene Oxide adsorbed unreacted hairpins, effectively quenching their fluorescence which reduced the signal background [16]. The 2 HCR hairpins were then combined and added to the MIST array overnight at RT. Following overnight incubation, the MIST array followed several rounds of washing with HCR amplification buffer before proceeding to protein detection.

To detect protein signals on the MIST array starting with amine beads, the array was incubated with a solution composed of 10 μg/mL streptavidin (Invitrogen, Waltham, MA, USA) and 3% bovine serum albumin (BSA, procured from Sigma-Aldrich, St. Louis, MO, USA) in PBS for 15 min. Biotinylated antibody (R&D Systems, Minneapolis, MN, USA) was then added at a 1:10 dilution for 15 min followed by three washes with PBS. Next, streptavidin-Alexa Fluor 647 (Invitrogen, Waltham, MA, USA) was added to the array for 15 min. The array slide underwent five rinses in the same buffer prior to imaging and scanning on a fluorescent microscope.

### 2.4. MIST Array Decoding Process

Following the detection process, the entire MIST array was scanned using a Nikon Ti2 inverted fluorescence microscope equipped with a motorized stage. For carboxyl-bearing arrays, a 40× objective with a high numerical aperture (NA) was used to capture the protein signals present on each microbead. A 20x objective was used for amine-bearing MIST arrays. The MIST arrays were then treated with 1 M NaOH solution for 2 min to facilitate the disassociation of the double-stranded DNAs. This was succeeded by a thorough wash with saline-sodium citrate (SSC) buffer five times. Subsequently, a mixture of cDNAs tagged with fluorophores at a concentration of 200 nM in a hybridization buffer (constituted by 40% formamide and 10% dextran sulfate in SSC buffer) was added to the MIST array and incubated for 1 h at ambient temperature. The array was then washed three times with SSC buffer and imaged using a fluorescence microscope, marking the conclusion of “Cycle 1” of the decoding process. The array was scanned in a manner identical to that utilized for protein detection.

The second and, if necessary, third cycles termed “Cycle 2” and “Cycle 3” were conducted in a similar fashion but with different combinations of cDNA-fluorophores. For the detection of nine proteins, decoding was accomplished in only two cycles. All images captured from protein detection to decoding cycles were aligned to determine the sequence of fluorescent color alterations for each microbead [17]. The sequence was pre-established for every category of oligo DNA microbead or protein type (see Table S2).

### 2.5. MIST Array Data Acquisition and Image Registration

A high-contrast, bright field image was captured in every section using a Nikon Ti2 inverted fluorescence microscope equipped with a motorized stage to pinpoint the positions of individual microbeads. The image suite for each cycle’s decoding included automatic capturing of fluorescence signals from Alexa Fluor 488, Cy3, Cy5 and Alexa Fluor 750 via a wavelength switcher. The entire microarray surface area was imaged in this fashion, frame by frame. The Nikon software (NIS-Elements AR Microscope Imaging Software) was used to save each individual image into 16-bit ND2 format, after which they were subjected to additional processing and registration through MATLAB programs developed in the lab. The programs use bright field images as the reference to align all the beads in protein detection and decoding cycles together and then assign a protein identity to each bead. The estimation of image registration error was conducted by gauging the average translational distance of corresponding points that underwent improper transformation during registration. Corresponding points were characterized as pixels with matching intensity values in both the static and the transformed images. In an ideal registration scenario, these points should remain stationary post-registration. The average displacement of all corresponding points was computed for every decoding cycle across all images.

To find the protein expression profiles of single cells, images from the cell marker channel were analyzed by CellProfiler to segment individual cells. The beads within a cell segment will be spatially identified, and their protein expression profiles will be recorded. The maximum protein intensity is taken as the protein expression level if multiple beads corresponding to the same protein are found within a cell segment. To generate pseudocells, the images were segmented into 12 × 12 μm^2^ units across the entire microarray. The protein expression of beads within each pseudocell was collected to generate a full profile of 9 proteins.

### 2.6. Data Processing and Statistics

The single-cell data and the pseudocell data were first filtered before bioinformatic analysis. The data were subtracted by the background plus two standard deviations as the threshold to obtain the true signal and protein expression. The single-cell data were further filtered by excluding those with low lamin B expression using CellProfiler’s minimum cross-entropy thresholding method. A lower bound of 0.2 was set for the intensity threshold in order to exclude all foreign objects as well as cells with very low intensities. Cells that were excluded might have lost the majority of their cell body during the sectioning process. For pseudocells, those with <20% detectable proteins were excluded in further data processing. All the data were processed by log2 and z-score normalization before clustering. We employed Uniform Manifold Approximation and Projection (UMAP) for reducing dimensionality and visualizing single cells and pseudocell clusters through a Seurat package in R. In the preprocessing phase, Principal Component Analysis (PCA) was applied to eliminate substantial outliers. The subpopulations were grouped by unsupervised UMAP clustering, and the markers in each subpopulation were identified. The expression levels of marker proteins were plotted in a heatmap. An unpaired, 2-tailed T-test was used to evaluate the statistical significances between mean surface area of cells vs. printed cells with Excel where a *p* value < 0.05 indicated a significant difference between groups.

## 3. Results and Discussion

**Figure 1:**
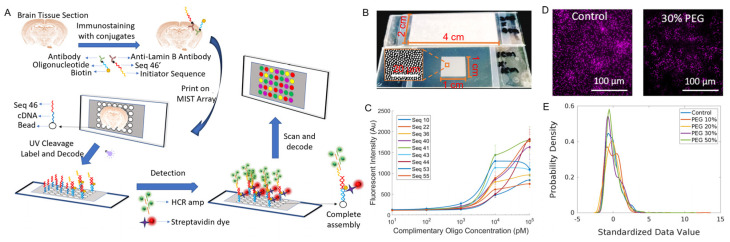
Overview of spatial MIST. (**A**) Schematic of the spatial MIST technique and the detection procedure. (**B**) Images of various sizes of MIST arrays on a glass slide. (**C**) Sensitivity of spatial MIST array for multiplexed detection of 9 DNA sequences. Calibration curves were generated by adding various concentrations of cDNA tagged with Alexa Fluor 647 and measuring microbead intensity at those conditions. (**D**) Detection of Fox−3 protein by spatial MIST under various concentrations of 8-arm PEG. Left: control; right: 30% PEG. Scale bar at 100 μm. (**E**) Intensity distribution curves of microbeads from MIST arrays under various concentrations of PEG.

The spatial MIST technique has been significantly improved in this report, including co-localization of cells and protein detection on the same array, single-cell resolution and iterative staining to achieve a multiplexity over 100. This technique generally comprises a monolayer array of microbeads carrying oligonucleotide barcodes and custom-made UV-cleavable cDNA-antibody conjugates. A tissue section is stained with the cDNA-antibody conjugates and is mated with the spatial MIST array. Upon UV exposure, the cDNAs are released from conjugates and captured locally by the MIST beads through diffusion (Figure 1A). In this way, multiplexed protein detection is converted from multiplexed DNA sensing. A decoding process is followed to identify the type of protein detected on the MIST array beads. We demonstrate in this report that the whole detection process can be repeated with different conjugates to reach ultrahigh multiplexity.

The performance of the spatial MIST technique has been fully characterized to reach single-cell resolution. Since tissue sections could be in a wide range of sizes, we show that the array can be varied from hundreds of micrometers to 2 cm × 4 cm (Figure 1B). The sensitivity of the MIST array was assessed by measuring the fluorescence signals of various concentrations of cDNAs (10 pM, 100 pM, 1 nM, 10 nM and 100 nM) in a solution designed to simulate cDNAs released from the tissue (Figure 1C). The detection limit was quantitated in a range between 36 and 130 pM. Compared with our previous studies using 2 μm beads, the sensitivity is slightly lower because the smaller beads carry a fewer number of oligos on each of them. Two UV cleavable linkers were designed between the oligo cDNAs and the antibodies, and over 99% of cDNA could be released within 5 min of UV exposure [18]. Since cDNAs are released and diffuse to the mated microarray, spatial resolution would be severely influenced by the medium and the diffusion distance. We found that 8-arm PEG is the most effective polymer to curb cDNA diffusion compared with linear PEG. We tested various concentrations of 8-arm PEG, specifically 10%, 20%, 30% and 50%, and employed one of ten types of MIST beads to detect the Fox-3 signal. Generally, with increasing PEG concentration, the cellular shape becomes more distinct; however, there is a concomitant gradual decrease in the fluorescence intensities of the beads (Figure 1D and Figure S2). The histogram of Figure 1E shows the distribution of bead intensity over PEG concentrations. The 30% PEG condition exhibits a considerably more uniform distribution compared to those with lower PEG concentrations, and its quantified intensity is greater than that of the 50% PEG condition. Thus, 30% PEG was used in all the subsequent MIST experiments.

**Figure 2:**
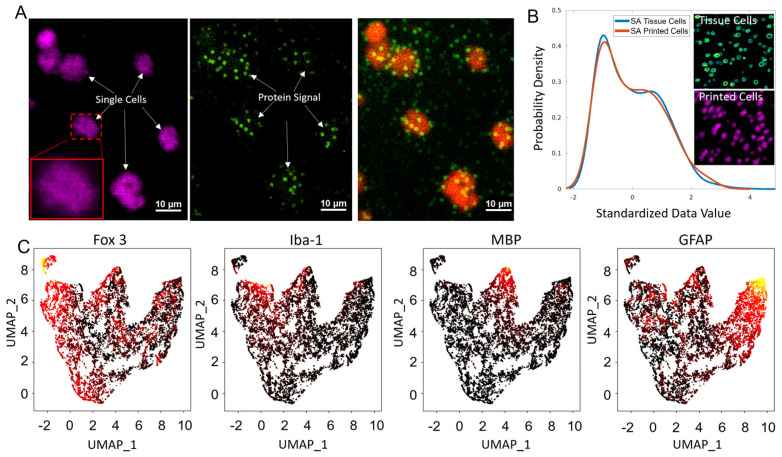
Detection of 9 proteins by single−cell spatial MIST. (**A**) Images of spatial MIST array in two fluorescent channels. Lamin B signal after HCR amplification and other protein signals on the MIST array are on the left and middle images, which are overlaid on the right image. Scale bar: 10 μm. (**B**) Comparison of the surface area distribution of printed cells (Lamin B signal) on the MIST array and tissue cells. (**C**) UMAP clustering of single cells shows 4 distinct clusters using an inferno colormap.

Single-cell resolution of the spatial MIST technique is achievable with the current enhancement of the detection strategy. Small microbeads at 1.4 µm are used to minimize the size of the MIST array area with a complete protein profile. Each microbead carries dual DNA sequences where one section is used to detect lamin B to locate cell location and the other section is designated to measure nine protein types simultaneously. Lamin B is a nuclear marker found in most brain cells through staining, and this way cells can be directly located on the MIST array [19,20,21]. In order to visualize the lamin B signal and cell nuclei on the array, we utilized hybridization chain reaction (HCR) to specifically amplify the fluorescence signal associated with it. The cDNA used for lamin B detection terminates with an initiator sequence for HCR amplification. Conversely, the cDNAs employed for the multiplex detection of the nine proteins, ends with biotin and they are labeled with a fluorescence color distinct from the HCR-amplified signals. Figure 2A shows images of single cells “printed” on the MIST array and the corresponding Fox-3 signal which appears to overlap with cells. Although nuclear staining is used in this report for the convenience of computational analysis, further study can rely on membrane markers which may better define cell boundaries. The influence of diffusion on signal detection is negligible (Figure 2B). Cell sizes on the tissue and the MIST array are measured to be 570.9 µm^2^ and 568.2 µm^2^, respectively, which show no significant differences (*p* < 0.05). The cell size distributions are also highly similar.

The single-cell spatial MIST has been demonstrated to detect nine critical protein markers of mouse brain tissue. The cells were clustered by UMAP to identify subpopulations by four proteins—Fox-3, Iba-1, Myelin Basic Protein (MBP) and Glial Fibrillary Acidic Protein (GFAP) (Figure 2C). Fox-3 acts as a neuronal marker, targeting the nuclei of neurons, whereas GFAP is a protein found in high abundance in astrocytes [22,23,24,25]. MBP is typically present in oligodendrocytes and Schwann cells, while Iba-1 is specifically associated with microglia [26,27,28,29]. Consistent with expectation, Fox-3 and GFAP expressing cells have larger populations than the other two. Consequently, our UMAP results are in alignment with existing knowledge about these proteins, validating our spatial MIST technique as a reliable method for analyzing protein expression in single cells.

**Figure 3:**
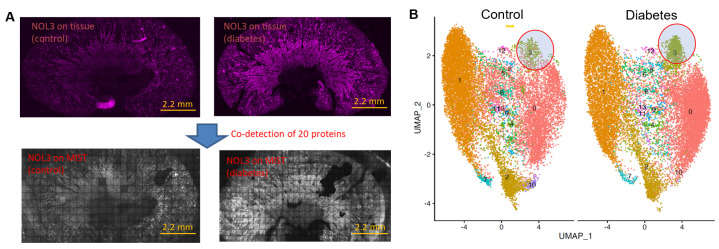
Co−detection of 20 proteins in kidney sections. (**A**) Immunostaining images of NOL3 on control kidney section (top left) and diabetic kidney section (top right). NOL3 detection on MIST array in control kidney section (bottom left) and diabetic kidney sections (bottom right). Scale bar: 2.2 mm. (**B**) UMAP clustering of pseudocells identified distinct clusters. Clusters 3, circled in red, appears to be denser for the diabetic kidney section.

The application of spatial MIST is not limited to brain tissue samples. Figure 3 demonstrates the multiplex analysis of mouse kidney sections by spatial MIST. Since kidney sections include a very significant portion of non-cell areas, we resorted to pseudocell strategy to profile the molecular distribution across the entire sections. The size of pseudocells has been set at 10 μm so they are almost the same size as cells. We examined 20 proteins on two kidney sections, one from a diabetic mouse (db/db) and another from an age-matched wildtype mouse (db/+). It was found that NOL3 has very different expression levels between the wildtype and the diabetic kidneys (Figure 3A). The NOL3 protein, also known as Apoptosis Repressor with CARD Domain (ARC), is involved in inhibiting apoptosis, or programmed cell death, and is expressed in a variety of human tissues [30,31]. The reconstructed images displaying NOL3 expression on MIST arrays reveal distributions and levels of expression that are consistent with those observed in the immunostained tissue images. The pseudocells were clustered and separated by UMAP. Cluster 3 highlighted in Figure 3B exhibited increased NOL3 expression in the diabetic kidney section compared to the wildtype kidney section, as many more pseudocells are observed in this cluster (Figure 3B). This elevation could be attributed to enhanced apoptotic pathway signaling in diabetic kidney disease, which is believed to contribute to the progression of the disease pathology [32,33,34].

**Figure 4:**
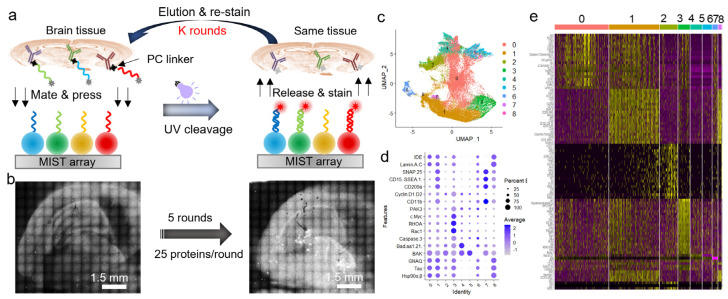
Detection of 125 proteins on a mouse coronal section using an iterative method. (**a**) Schematic of multi−round conjugate staining and MIST protein detection. (**b**) Scanned images of MIST array after the 1st round of spatial MIST detection (left) and after the 5th round of spatial MIST detection (right). (**c**) UMAP clustering of pseudo cells identifies 9 distinct clusters. (**d**) Dot plots of distinctive proteins over identified groups. (**e**) Heatmap of top proteins expressed in different groups of pseudocells.

We further demonstrate the ability of spatial MIST in the detection of over a hundred proteins through an iterative method. The protein panel is selected only for the purpose of demonstration. As shown in Figure 4a, a mouse brain section was stained with 25 DNA-antibody conjugates per round and printed on a MIST array; the same tissue section can be eluted and processed by the same detection process a few times. Each round co-detected 25 proteins on a separate MIST array, and all the MIST arrays were aligned so that each pseudocell corresponds to the same tissue location across all 5 rounds. Figure 4b shows the MIST array images of the first round and the last round, each with 25 proteins co-detected. All the pseudocells with 125 protein profiles were clustered by the Seurat package to identify 9 distinctive subtypes (Figure 4c). Since the 125 proteins were not specifically selected for brain cells and collectively represent only a segment of the cellular system, the subtypes identified through unsupervised clustering are distinguished solely within the context of this 125-protein defined space. It is worth noting that the pseudocells include non-cellular areas; as such, these pseudocells enable the quantification of the intercellular microenvironment. The dot plot in Figure 4d discovered the most significant proteins in each cluster. The program identified the top proteins for each cluster shown in a heatmap (Figure 4e). This method will be highly informative for mechanistic studies if the disease-relevant proteins and samples are used in the future.

## 4. Conclusions

In summary, we have developed a single-cell spatial MIST technology to analyze protein markers from various tissue sources with a multiplexity from 9 to 125. The performance of the technology has been fully characterized to reach the single-cell resolution. It was found that an 8-arm PEG with 30% concentration can significantly improve the spatial resolution while keeping the short detection time. The use of small microbeads at 1.4 µm diameter for fabrication of MIST arrays further increases the spatial resolution as cell nuclei labeled by lamin B were clearly visualized on the arrays. These measures are crucial for minimizing signal contamination from cells that are in close proximity within the same tissue. The dual sequence on each MIST microbead enables colocalization of the lamin B signal and the 9-protein detection signal, which enables a comprehensive profiling of protein expression levels within tissue cells. Since tissue often includes a large portion of non-cell regions and intercellular spaces, pseudocells that are set at 10 μm by 10 μm squares can better represent all tissue features including the intercellular microenvironment. Application of spatial MIST on kidney sample sections reveals NOL3 differential expression between wildtype and diabetic mice. We also demonstrate the ultrahigh multiplexity capability of spatial MIST through iterative staining and detection on the same tissue section. Each round detects 25 proteins, and all 5 rounds are aligned to reach 125 proteins for every pseudocell. Through bioinformatic analysis, 9 clusters have been discovered and the significant proteins expressed in those clustered have also been distinguished. The robust methodology presented in this report sets the foundation for future mprehensive biological investigations across diverse tissue types.

## Data Availability

Data available upon request.

## Supplementary Materials

The following supporting information can be downloaded at: https://www.mdpi.com/article/10.3390/bios13090852/s1. Figure S1: Images of microarrays 1.4 μm and 2 μm. Figure S2: Effects of varying Concentrations of 8-arm PEG added before UV cleavage on Spatial MIST array. Table S1: List of all antibodies and oligonucleotide sequences used. Table S2: Color code scheme of each experiment is tabulated.
