# Supplementary material for: Single-Cell Spatial MIST for Versatile, Scalable Detection of Protein Markers
Source: Biosensors (Basel). 2023 Aug 27;13(9):852. doi: 10.3390/bios13090852 (PMC10526469; doi:10.3390/bios13090852)
Supplement: Supplementary file 1 [file biosensors-13-00852-s001.zip › biosensors-2549409-supplementary.pdf]

Supplementary Information

Single-Cell Spatial MIST for Versatile, Scalable Detection of Protein Markers

Arafat Meah [1], Vadanasundari Vedarethinam [1], Robert Bronstein [2], Nehaben Gujarati [2], Tanya Jain [3,4], Sandeep K. Mallipattu [2,5], Yueming Li [3,4,6] and Jun Wang [1,*]

1. Multiplex Biotechnology Laboratory, Department of Biomedical Engineering, State University of New York at Stony Brook, Stony Brook, NY 11794, USA
2. Division of Nephrology and Hypertension, Department of Medicine, Stony Brook School of Medicine, Stony Brook, NY 11794, USA
3. Chemical Biology Program, Memorial Sloan Kettering Cancer Center, New York, NY 10065, USA
4. Programs of Neurosciences, Weill Graduate School of Medical Sciences of Cornell University, New York, NY 10065, USA
5. Renal Section, Northport VA Medical Center, Northport, NY 11768, USA
6. Programs of Pharmacology, Weill Graduate School of Medical Sciences of Cornell University, New York, NY 10021, USA

* Correspondence: jun.wang.5@stonybrook.edu

Table S1: The following tables are a list of antibodies and oligonucleotides used in this work.

**Primary antibody**				
**Protein marker**	**Vendor**	**Catalog #**	**Isotype**	**Clone**
GAPDH	Biolegend	607902	Rat IgG2a	W17079A
Phospho-PLCγ1 (Tyr783) (p-PLCγ1)	Biolegend	612402	Mouse IgG1	A17025A
VGAT	Invitrogen	MA5-24643	Mouse IgG1	CL2793
β-actin	Biolegend	643802	Mouse IgG2b	2F1-1
Hsp70	Biolegend	648002	Mouse IgG2a	W27
LC3	Biolegend	848802	Mouse IgG2b	A15143K
Akt	R&D Systems	MAB2055	Mouse IgG2B	281046
Nicastrin	Biolegend	852302	Mouse IgG2b	9C3
FOX3	Biolegend	834501	Mouse IgG2b	1B7
NMDAR1	Invitrogen	32-0500	Mouse IgG2a	54.1
Bcl-2	Biolegend	633502	Mouse IgG1	BCL/10C4
Drebrin	Invitrogen	PA5-100142	Rabbit IgG	Not applicable

Amyloid Precursor Protein (APP)	Invitrogen	13-0200	Mouse IgG1	LN27
HSF1	Biolegend	825801	Rat IgG2a	4B4
Phospho mTOR (Ser2448) (p-mTOR)	Biolegend	610302	Mouse IgG1	A17024A
Phospho STAT3 (Tyr705) (p-STAT3)	Biolegend	651002	Mouse IgG1	13A3-1
Phospho ERK1/2 (Thr202/Tyr204) (p-ERK1/2)	Biolegend	675502	Mouse IgG2b	4B11B69
Phospho Lck (Tyr394) (p-Lck)	Biolegend	933102	Mouse IgG1	A18002D
Neurofilament H&M (NF H&M)	Biolegend	837704	Mouse IgG1	SMI 310
CD24	Biolegend	101802	Rat IgG2b	M1/69
CD304	Biolegend	145202	Rat IgG2a	3E12
Pax-5	Biolegend	649702	Rat IgG2a	1H9
SOX2	Biolegend	656102	Mouse IgG1	14A6A34
Tubulin β3 (Tubulin)	Biolegend	801201	Mouse IgG2a	TUJ1
MAP2	Biolegend	801801	Mouse IgG1	SMI52
NSE	Biolegend	804901	Mouse IgG1	NSE-P1
MAGUK	Biolegend	817801	Mouse IgG1	K28/86
Neuroligin-1	Biolegend	819001	Mouse IgG1	N97A/31
VGlut1	Biolegend	821301	Mouse IgG1	N28/9
Dopamine Transporter (DAT)	Invitrogen	PA5-106751	Rabbit IgG	Not applicable
UFD1	Biolegend	671302	Rat IgG2a	W18140A
CD11b	Biolegend	101247	Rat IgG2b	M1/70
CD16/32	Biolegend	101302	Rat IgG2a	93
CD40	Biolegend	102802	Rat IgG2a	1C10
CD45	Biolegend	103102	Rat IgG2b	30-F11
CD68	Biolegend	137001	Rat IgG2a	FA-11
CD86	Biolegend	105002	Rat IgG2a	GL-1
I-A/I-E	Biolegend	107602	Rat IgG2b	M5/114.15.2
CD206	Biolegend	141701	Rat IgG2a	C068C2
CD301a	Biolegend	145602	Rat IgG2a	LOM-8.7
CD163	Biolegend	155302	Rat IgG2a	S15049I

CD15	Biolegend	125602	Mouse IgM	MC-480
CD115	Biolegend	135502	Rat IgG2a	AFS98
CD64	Biolegend	139301	Mouse IgG1	X54-5/7.1
CD209a	Biolegend	833001	Mouse IgG2c	MMD3
SNAP-25	Biolegend	836304	Mouse IgG1	SMI 81
Myelin Basic Protein (MBP)	Biolegend	836504	Mouse IgG1	SMI 94
Synaptophysin	Biolegend	837104	Mouse IgM	SP17
GFAP	Biolegend	837404	Mouse IgG2b	SMI 24
ATG5	Biolegend	847402	Mouse IgG1	177.19
SynDIG1	Biolegend	851802	Mouse IgG2a	L42/17
Shank	Biolegend	851902	Mouse IgG1	N23B/49
TFEB	Biolegend	852002	Mouse IgG2b	A17106C
ALDH1L1	Biolegend	856802	Mouse IgG1	N103/39
Arl13b	Biolegend	857602	Mouse IgG2a	N295B/66
UBE2O	Biolegend	870202	Mouse IgG2b	A18140A
E1 Ubiquitin Activating Enzyme (UBE1)	Biolegend	932002	Mouse IgG1	2G2.3.5
GABRB3	Biolegend	818501	Mouse IgG1	N87/25
ERK1/2	Biolegend	686902	Rat IgG1	W15133B
Glutamine Synthetase	Invitrogen	701989	Rabbit IgG	7H9L16
GLT-1	Invitrogen	701988	Rabbit IgG	9H9L17
CD56	Invitrogen	701379	Rabbit IgG	3H15L12
S100B	Invitrogen	710363	Rabbit IgG	16HCLC
MEK	Invitrogen	13-3500	Mouse IgG1	3D9
Lamin B1	Biolegend	869802	Mouse IgG2b	5G8-D3-H7

CREB	Invitrogen	35-0900	Mouse IgG1	LB9
VGLUT2	Invitrogen	42-7800	Rabbit IgG	Not applicable
NG2	Invitrogen	MA5-24247	Rat IgG1	546930
GABRG1	Invitrogen	PA5-99317	Rabbit IgG	Not applicable
Tyrosine Hydroxylase	Invitrogen	PA5-85167	Rabbit IgG	Not applicable
SLC6A1	Invitrogen	PA5-85766	Rabbit IgG	Not applicable
TLR4	Invitrogen	710185	Rabbit IgG	20HCLC
Phospho-Tau (Ser199) (p-Tau)	Invitrogen	701054	Rabbit IgG	2H23L4
TrkC	Invitrogen	701985	Rabbit IgG	7H3L20
Phospho-IRS1 (Ser312) (p-IRS1)	Invitrogen	710778	Rabbit IgG	18HCLC
Cofilin	Invitrogen	MA5-17275	Mouse IgG2a	GT567
Acetylcholinesterase (AChE)	Invitrogen	MA3-042	Mouse IgG2b	HR2
Clathrin	Invitrogen	MA1-065	Mouse IgG1	X22
Adaptin	Invitrogen	MA3-061	Mouse IgG2a	AC1-M11
PLP1	Invitrogen	PA5-40788	Rabbit IgG	Not applicable
VAChT	Invitrogen	MA5-27662	Mouse IgG1	S6-38
SOX10	Invitrogen	703439	Rabbit IgG	5H7L26
CaMKII	Invitrogen	MA1-048	Mouse IgG1	6G9
COX1	Invitrogen	35-8100	Mouse IgG2b	COX 111
Oligodendrocyte Marker O1 (O1)	Invitrogen	14-6506-82	Mouse IgM	O1
SHP2	Invitrogen	PA5-20279	Rabbit IgG	Not applicable
CaV1.2	Invitrogen	MA5-27717	Mouse IgG1	S57-46
LDLR	Invitrogen	PA5-46987	Goat IgG	Not applicable

CDC42	Proteintech	10155-1-AP	Rabbit IgG	Not applicable
TRADD	Invitrogen	PA5-106869	Rabbit IgG	Not applicable
Phospho-INSR (Tyr1361) (p-INSR)	Invitrogen	PA5-104754	Rabbit IgG	Not applicable
Phospho-RHOA (Ser188) (p-RHOA)	Invitrogen	PA5-105763	Rabbit IgG	Not applicable
HMGCS1	Invitrogen	PA5-100011	Rabbit IgG	Not applicable
Phospho-Amyloid Precursor Protein (Thr668) (p-APP)	Invitrogen	PA5-104745	Rabbit IgG	Not applicable
ASAH1	Invitrogen	PA5-20574	Rabbit IgG	Not applicable
Phospho-Calmodulin 1/2/3 (Thr79, Ser81) (p-Calmodulin)	Invitrogen	PA5-104932	Rabbit IgG	Not applicable
Phospho-SREBP1 (Ser439) (p-SREBP1)	Invitrogen	PA5-99371	Rabbit IgG	Not applicable
COX2	Proteintech	12375-1-AP	Rabbit IgG	Not applicable
TrkA	Invitrogen	PA5-98018	Rabbit IgG	Not applicable
Dynamin 1	Invitrogen	PA5-91865	Rabbit IgG	Not applicable
CHRFAM7A	Invitrogen	PA5-96829	Rabbit IgG	Not applicable
HMGCR	Invitrogen	PA5-95846	Rabbit IgG	Not applicable
GSK-3 alpha / beta (GSK-3)	R&D Systems	AF2157	Rabbit IgG	Not applicable
Mu-Calpain	Invitrogen	PA5-27681	Rabbit IgG	Not applicable
TrkB	Invitrogen	PA5-78405	Rabbit IgG	Not applicable
FOXO1	Invitrogen	MA5-17078	Mouse IgG1	3B6
APOE	Invitrogen	701241	Rabbit IgG	16H22L18
PAK1	Invitrogen	71-9300	Rabbit IgG	Not applicable
TREM2	Invitrogen	MA5-31267	Rabbit IgG	14
Phospho-CREB/ATF1 (Ser133, Ser63) (p-CREB/ATF1)	Invitrogen	MA1-114	Mouse IgG1	10E9
LEF1	Invitrogen	PA5-102851	Rabbit IgG	Not applicable

Phospho-JNK1/JNK2 (Thr183, Tyr185) (p-JNK1/JNK2)	Invitrogen	700031	Rabbit IgG	D12H7L17
PDCD4	Invitrogen	702494	Rabbit IgG	1H15L6
EIF2S1	Invitrogen	AHO0802	Mouse IgG1	EIF2-alpha
K-Ras	Invitrogen	415700	Mouse IgG1	9.13
Arp2	Invitrogen	703394	Rabbit IgG	5H2L7
Phospho-BRAF (Ser729) (p-BRAF)	Invitrogen	PA5-105501	Rabbit IgG	Not applicable
CK1 gamma-1	Invitrogen	PA5-104268	Rabbit IgG	Not applicable
Tau	Invitrogen	AHB0042	Mouse IgG1	TAU-5
NLK	Invitrogen	PA5-102708	Rabbit IgG	Not applicable
Phospho MEK1 (Ser298) (p-MEK1)	Biolegend	610602	Mouse IgG1	A16117B
Phospho-p70 S6 Kinase (Ser424) (p-p70 S6 Kinase)	Invitrogen	PA5-104844	Rabbit IgG	Not applicable
Phospho-beta Catenin (Thr41, Ser45) (p-beta Catenin)	Invitrogen	PA5-104873	Rabbit IgG	Not applicable
Phospho-BAD (Ser155) (p-BAD)	Invitrogen	PA5-105022	Rabbit IgG	Not applicable
GNAQ	Invitrogen	PA5-100253	Rabbit IgG	Not applicable
Phospho-TAK1 (Thr187) (p-TAK1)	Invitrogen	PA5-99340	Rabbit IgG	Not applicable
Phospho-SHP2 (Tyr62) (p-SHP2)	Invitrogen	PA5-105618	Rabbit IgG	Not applicable
MRLC2	Invitrogen	PA5-38295	Rabbit IgG	Not applicable
Caspase 9 (Cleaved Asp330)	Invitrogen	PA5-105272	Rabbit IgG	Not applicable
Phospho-PKA alpha (Ser338) (p-PKA alpha)	Invitrogen	PA5-105760	Rabbit IgG	Not applicable
BID	Invitrogen	PA5-11384	Rabbit IgG	Not applicable
AXIN1	Invitrogen	PA5-21042	Rabbit IgG	Not applicable
GLUT4	Invitrogen	MA5-17176	Mouse IgG2b	3G10A3
Hsp90α/β	Biolegend	661802	Mouse IgG1	K3720A

Lamin A/C	Invitrogen	PA5-78042	Rabbit IgG	Not applicable
CDK5	Invitrogen	PA5-96409	Rabbit IgG	Not applicable
ROCK1	Invitrogen	PA5-22262	Rabbit IgG	Not applicable
ERAB	Invitrogen	PA5-27155	Rabbit IgG	Not applicable
IDE	Invitrogen	PA5-79435	Rabbit IgG	Not applicable
PKC alpha	Invitrogen	PA5-87400	Rabbit IgG	Not applicable
JAK3	Invitrogen	PA5-87500	Rabbit IgG	Not applicable
APC	Invitrogen	PA5-96213	Rabbit IgG	Not applicable
Phospho-EGFR (Ser1047) (p-EGFR)	Invitrogen	PA5-36801	Rabbit IgG	Not applicable
GNAI3	Invitrogen	PA5-76885	Rabbit IgG	Not applicable
Phospho-Akt (S473) (p-Akt)	R&D Systems	AF887	Rabbit IgG	Not applicable
Phospho-STAT1 (Y701) (p-STAT1)	R&D Systems	AF2894	Rabbit IgG	Not applicable
HSP60	Biolegend	681502	Mouse IgG1	P83G8
TOR	R&D Systems	MAB1537	Rat IgG	303728
Notch-1	R&D Systems	MAB5267	Mouse IgG1	N1A
Notch-2	R&D Systems	MAB5196	Rat IgG	605724
Notch-3	R&D Systems	AF1308	Goat IgG	Not applicable
Phospho-GSK-3 alpha/beta (S21/S9) (p-GSK3)	R&D Systems	AF1590	Rabbit IgG	Not applicable
INSR	Invitrogen	PA5-87344	Rabbit IgG	Not applicable
PDK-1	R&D Systems	MAB864	Mouse IgG2	650308
PTEN	R&D Systems	AF847	Rabbit IgG	Not applicable
eIF4E	R&D Systems	MAB3228	Mouse IgG2	299910
Src	R&D Systems	AF3389	Goat IgG	Not applicable

TSC1	R&D Systems	AF4379	Sheep IgG	Not applicable
TSC2	R&D Systems	MAB4040 1	Mouse IgG1	614204
Rheb	R&D Systems	MAB3426	Mouse IgG2	344912
GRB2	R&D Systems	AF3846	Goat IgG	Not applicable
STAT5a	R&D Systems	MAB2174	Mouse IgG3	251619
STAT5b	R&D Systems	AF1584	Rabbit IgG	Not applicable
Jak1	R&D Systems	MAB4260	Rat IgG	413104
Beclin 1	Invitrogen	PA5-20171	Rabbit IgG	Not applicable
Cyclin D1/D2	R&D Systems	AF4196	Goat IgG	Not applicable
MDM2/HDM2	R&D Systems	AF1244	Rabbit IgG	Not applicable
Active Caspase-3	R&D Systems	AF835	Rabbit IgG	Not applicable
BAD	R&D Systems	AF819	Rabbit IgG	Not applicable
PAK3	Invitrogen	PA5-112638	Rabbit IgG	Not applicable
p53	R&D Systems	MAB1355	Mouse IgG2	184721
RelA/NF kappa B p65	R&D Systems	MAB5078	Mouse IgG2	532301
cIAP-2/HIAP-1	R&D Systems	MAB817	Mouse IgG1	315304
Oligodendrocyte Marker O4 (O4)	R&D Systems	MAB1326	Mouse IgM	O4
RHOA	Proteintech	10749-1-AP	Rabbit IgG	Not applicable
TNFR1	Proteintech	60192-1-IG	Mouse IgG1	2A6E3
Rac1	Proteintech	66122-1-IG	Mouse IgG2b	4A4B11
NOL3	Proteintech	10846-2-AP	Rabbit IgG	Not applicable
PI3K p85 alpha	Proteintech	60225-1-IG	Mouse IgG2a	4G3C11
4EBP1	Proteintech	60246-1-IG	Mouse IgG2b	2C3F3

SIRT1	Proteintech	60303-1-IG	Mouse IgG2b	7F6G6
JNK	Proteintech	66210-1-IG	Mouse IgG1	1A12E1
Iba-1	Invitrogen	PA5-27436	Rabbit / IgG	Not applicable
beta Amyloid	Invitrogen	36-6900	Rabbit / IgG	Not applicable

**Secondary antibody**				
**No.**	**Antibody name**	**Vendor**	**Catalog #**	**Fluorophore**
1	Goat anti-Mouse IgG secondary antibody	Invitrogen	A-11001	Alexa Fluo 488
2	Goat anti-Rat IgG secondary antibody	Invitrogen	A-11006	Alexa Fluo 488
3	Goat anti-Rabbit IgG secondary antibody	Invitrogen	A-11008	Alexa Fluo 488
4	Goat anti-Mouse IgG secondary antibody	Invitrogen	A-21235	Alexa Fluo 647
5	Goat anti-Mouse IgM secondary antibody	Invitrogen	A-21238	Alexa Fluo 647
6	Goat anti-Rat IgG secondary antibody	Invitrogen	A-21247	Alexa Fluo 647
7	Goat anti-Rabbit IgG secondary antibody	Invitrogen	A-21244	Alexa Fluo 647
8	Donkey anti-Goat IgG secondary Antibody	Invitrogen	A-21447	Alexa Fluo 647
9	Donkey anti-Sheep IgG Secondary Antibody	Invitrogen	A-21448	Alexa Fluo 647

Oligonucleotide Sequences

The following oligonucleotide sequences were used in order to modify the microbeads in our work.

Sequence Name	Sequence 5′→3′
Seq1	/5AmMC6/ATG TCT TCC GGC GCA
Seq2	/5AmMC6/AAT GAG TGC GCG GTC
Seq3	/5AmMC6/GGA TTG ACG CAG GCA
Seq4	/5AmMC6/CAA GAG CGC CGA ACT
Seq5	/5AmMC6/AGT TGG TGC CTG GTG
Seq6	/5AmMC6/CCG GCG AGT TCT GTT
Seq7	/5AmMC6/TCC GGC CAC TCT ACT

Seq8	/5AmMC6/ATG GTT GGA CGG AGC
Seq9	/5AmMC6/TTC GGC ACA CAG AGG
Seq10	/5AmMC6/TTC ACT CGT CGG CTG
Seq11	/5AmMC6/ACG ACG AAG CCA CTC
Seq12	/5AmMC6/TAT TCC ACC GCG ACC
Seq13	/5AmMC6/CGG AAC CAG GAA GCT
Seq14	/5AmMC6/AGA GTA CGC GCT TCC
Seq15	/5AmMC6/CCA GGT CAG CGA GTT
Seq16	/5AmMC6/GCA AGC ACT CGT TCG
Seq17	/5AmMC6/GCA GGC TAG GTC GAA
Seq18	/5AmMC6/ATC CAA GCG CGA GAA
Seq19	/5AmMC6/ACG CTC CTC CTC ATG
Seq20	/5AmMC6/AGT CTC TCG GCC ATG
Seq21	/5AmMC6/TTC AGT TCA CGG CCA
Seq22	/5AmMC6/CGC CGG TTG AGA TTG
Seq23	/5AmMC6/ATC ACT GCC GAC GAA
Seq24	/5AmMC6/AGC CAC TTC ACA CCA
Seq25	/5AmMC6/GAC CTC GTA CCA CCT
Seq26	/5AmMC6/GCT CTC GAC GTG TTC
Seq27	/5AmMC6/CTG GCG CTG TTG AAT
Seq28	/5AmMC6/CGA GTG CCA GTG ATC
Seq29	/5AmMC6/CTG ATC CGC CGT AAG
Seq30	/5AmMC6/CAC GGT GAG GAT GAC
Seq31	/5AmMC6/TCG GAT GGC AAT GTG
Seq32	/5AmMC6/CAA CGG ACG GAC ATT
Seq33	/5AmMC6/TCT GGC GCT AAC AAC
Seq34	/5AmMC6/GAG ACG GCG TAG ATG
Seq35	/5AmMC6/ACT ATG GCC TGG TCT
Seq36	/5AmMC6/TCA TAA GCT CGC GTC
Seq37	/5AmMC6/AGC GAA CAG GTG ATC
Seq38	/5AmMC6/ACA ACT CTA ACC GGC
Seq39	/5AmMC6/GTC GCC GTT CAT CTA
Seq40	/5AmMC6/ACC AGA TTA CGC CAG
Seq41	/5AmMC6/ATGGTCGAGATGTCAGAGTA
Seq42	/5AmMC6/ATTGACCAAACTGCGGTGCG
Seq43	/5AmMC6/TCTTCTAGTTGTCGAGCAGG
Seq44	/5AmMC6/TAATCTAATTCTGGTCGCGG
Seq45	/5AmMC6/GTGATTAAGTCTGCTTCGGC
Seq46	/5AmMC6/GTCGAGGATTCTGAACCTGT
Seq47	/5AmMC6/GTCCTCGCTTCGTCTATGAG

Seq48	/5AmMC6/CTTCGTGGCTAGTCTGTGAC
Seq49	/5AmMC6/TAAGCCAGTGTGTCGTGTCT
Seq50	/5AmMC6/GCGTGTGTGGACTCTCTCTA

**Dual Sequence Name**	**Sequence 5′→3′**
Seq 35- Seq 9	/5AmMC6/ACT ATG GCC TGG TCT TTC GGC ACA CAG AGG
Seq 35- Seq 19	/5AmMC6/ACT ATG GCC TGG TCT ACG CTC CTC CTC ATG
Seq 35- Seq 28	/5AmMC6/ACT ATG GCC TGG TCT CGA GTG CCA GTG ATC
Seq 35- Seq 31	/5AmMC6/ACT ATG GCC TGG TCT TCG GAT GGC AAT GTG
Seq 35- Seq 32	/5AmMC6/ACT ATG GCC TGG TCT CAA CGG ACG GAC ATT
Seq 35- Seq 33	/5AmMC6/ACT ATG GCC TGG TCT TCT GGC GCT AAC AAC
Seq 35- Seq 34	/5AmMC6/ACT ATG GCC TGG TCT GAG ACG GCG TAG ATG
Seq 35- Seq 38	/5AmMC6/ACT ATG GCC TGG TCT ACA ACT CTA ACC GGC
Seq 35- Seq 39	/5AmMC6/ACT ATG GCC TGG TCT GTC GCC GTT CAT CTA

The following sequences were used in order to modify the antibodies in our work

**Sequence Name**	**Sequence 5′→3′**
Seq1'	/5Biosg/TGC GCC GGA AGA CAT /3AmMO/
Seq2'	/5Biosg/GAC CGC GCA CTC ATT /3AmMO/
Seq3'	/5Biosg/TGC CTG CGT CAA TCC /3AmMO/
Seq4'	/5Biosg/AGT TCG GCG CTC TTG /3AmMO/
Seq5'	/5Biosg/CAC CAG GCA CCA ACT /3AmMO/
Seq6'	/5Biosg/AAC AGA ACT CGC CGG /3AmMO/
Seq7'	/5Biosg/AGT AGA GTG GCC GGA /3AmMO/
Seq8'	/5Biosg/GCT CCG TCC AAC CAT /3AmMO/
Seq9'	/5Biosg/CCT CTG TGT GCC GAA /3AmMO/
Seq10'	/5Biosg/CAG CCG ACG AGT GAA /3AmMO/
Seq11'	/5Biosg/GAG TGG CTT CGT CGT /3AmMO/
Seq12'	/5Biosg/GGT CGC GGT GGA ATA /3AmMO/
Seq13'	/5Biosg/AGC TTC CTG GTT CCG /3AmMO/

Seq14'	/5Biosg/GGA AGC GCG TAC TCT /3AmMO/
Seq15'	/5Biosg/AAC TCG CTG ACC TGG /3AmMO/
Seq16'	/5Biosg/CGA ACG AGT GCT TGC /3AmMO/
Seq17'	/5Biosg/TTC GAC CTA GCC TGC /3AmMO/
Seq18'	/5Biosg/TTC TCG CGC TTG GAT /3AmMO/
Seq19'	/5Biosg/CAT GAG GAG GAG CGT /3AmMO/
Seq20'	/5Biosg/CAT GGC CGA GAG ACT /3AmMO/
Seq21'	/5Biosg/TGG CCG TGA ACT GAA /3AmMO/
Seq22'	/5Biosg/CAA TCT CAA CCG GCG /3AmMO/
Seq23'	/5Biosg/TTC GTC GGC AGT GAT /3AmMO/
Seq24'	/5Biosg/TGG TGT GAA GTG GCT /3AmMO/
Seq25'	/5Biosg/AGG TGG TAC GAG GTC /3AmMO/
Seq26'	/5Biosg/GAA CAC GTC GAG AGC /3AmMO/
Seq27'	/5Biosg/ATT CAA CAG CGC CAG /3AmMO/
Seq28'	/5Biosg/GAT CAC TGG CAC TCG /3AmMO/
Seq29'	/5Biosg/CTT ACG GCG GAT CAG /3AmMO/
Seq30'	/5Biosg/GTC ATC CTC ACC GTG /3AmMO/
Seq31'	/5Biosg/CAC ATT GCC ATC CGA /3AmMO/
Seq32'	/5Biosg/AAT GTC CGT CCG TTG /3AmMO/
Seq33'	/5Biosg/GTT GTT AGC GCC AGA /3AmMO/
Seq34'	/5Biosg/CAT CTA CGC CGT CTC /3AmMO/
Seq35'	/5Biosg/AGA CCA GGC CAT AGT /3AmMO/
Seq36'	/5Biosg/GAC GCG AGC TTA TGA /3AmMO/
Seq37'	/5Biosg/GAT CAC CTG TTC GCT /3AmMO/
Seq38'	/5Biosg/GCC GGT TAG AGT TGT /3AmMO/
Seq39'	/5Biosg/TAG ATG AAC GGC GAC /3AmMO/
Seq40'	/5Biosg/CTG GCG TAA TCT GGT /3AmMO/
Seq41'	/5Biosg/TAC TCT GAC ATC TCG ACC AT/3AmMO/
Seq42'	/5Biosg/CGC ACC GCA GTT TGG TCA AT/3AmMO/
Seq43'	/5Biosg/CCT GCT CGA CAA CTA GAA GA/3AmMO/
Seq44'	/5Biosg/CCG CGA CCA GAA TTA GAT TA/3AmMO/
Seq45'	/5Biosg/GCC GAA GCA GAC TTA ATC AC/3AmMO/
Seq46'	/5Biosg/ACA GGT TCA GAA TCC TCG AC/3AmMO/
Seq47'	/5Biosg/CTC ATA GAC GAA GCG AGG AC/3AmMO/

Seq48'	/5Biosg/GTC ACA GAC TAG CCA CGA AG/3AmMO/
Seq49'	/5Biosg/AGA CAC GAC ACA CTG GCT TA/3AmMO/
Seq50'	/5Biosg/TAG AGA GAG TCC ACA CAC GC/3AmMO/
Seq 35'-Initiator	/5AmMC6/AGA CCA GGC CAT AGT AAG AGG AGG GCA GCA AAC GGG AAG AGT CTT CCT TTA CG

Table S2: The following tables are the color schemes used for each experiment. The complimentary sequences dyes are conjugated with the listed fluorophores. The exact proteins a microbead represents are based off how each beads changes color in each cycle based off the color schemes below.

Spatial MIST Decoding

cDNA	cDNA-dye cocktail 1	cDNA-dye cocktail 2	Protein Panel (9)
	Cycle1	Cycle2	
Seq9'	Cy5	Cy5	Fox3
Seq19'	Cy3	AF 488	beta Amyloid
Seq28'	Cy5	Cy7	MBP
Seq31'	Cy7	Cy5	Neurofilament H&M (NF H&M)
Seq32'	Cy7	Cy7	Neuroligin-1
Seq33'	AF 488	AF 488	p-APP
Seq34'	AF 488	Cy3	COX 2
Seq38'	Cy5	Cy3	Iba-1
Seq39'	Cy3	Cy3	GFAP

Kidney Decoding

cDNA	cDNA-dye cocktail 1	cDNA-dye cocktail 2	cDNA-dye cocktail 3	Protein Panel (20)
	Cycle1	Cycle2	Cycle3	
Seq26'	Cy3	Cy5	Cy3	Human/Mouse/Rat Jak1 Antibody
Seq27'	Cy5	Cy5	Cy5	Human/Mouse Beclin 1 Antibody
Seq28'	Cy5	Cy5	Cy7	Human/Mouse Calpain 2
Seq29'	Cy5	Cy7	AF488	Human/Mouse Granzyme B Antibody

**Seq31'**	**Cy5**	**Cy7**	**Cy5**	**Human/Mouse Cyclin D1/D2 Cross-reactive Antibody**
**Seq32'**	**Cy5**	**Cy7**	**Cy7**	**Human/Mouse/Rat MDM2/HDM2 Antibody**
**Seq33'**	**Cy5**	**AF488**	**AF488**	**Human/Mouse/Rat Active Caspase-3**
**Seq34'**	**Cy5**	**AF488**	**Cy3**	**Human/Mouse Bad aa 1-21 Antibody**
**Seq36'**	**Cy5**	**AF488**	**Cy7**	**Human/Mouse PAK3 Antibody**
**Seq37'**	**Cy5**	**Cy3**	**AF488**	**Human/Mouse/Rat p53 Antibody**
**Seq40'**	**Cy7**	**Cy3**	**Cy7**	**Human/Mouse/Rat RelA/NF kappa B p65 Antibody**
**Seq41'**	**Cy7**	**Cy5**	**AF488**	**Human/Mouse Phospho-STAT1 (Y701)**
**Seq42'**	**Cy7**	**Cy5**	**Cy3**	**Human/Mouse/Rat/Chicken Oligodendrocyte Marker O4 Antibody**
**Seq44'**	**Cy7**	**Cy5**	**Cy7**	**TNFR1 Monoclonal Antibody (2A6E3)**
**Seq45'**	**Cy7**	**Cy7**	**AF488**	**Rac1 Monoclonal Antibody (4A4B11)**
**Seq46'**	**Cy7**	**Cy7**	**Cy3**	**NOL3 Polyclonal Antibody**
**Seq47'**	**Cy7**	**Cy7**	**Cy5**	**PI3K p85 alpha Monoclonal Antibody**
**Seq48'**	**Cy7**	**Cy7**	**Cy7**	**4EBP1 Monoclonal Antibody (2C3F3)**
**Seq49'**	**Cy7**	**AF488**	**AF488**	**SIRT1 Monoclonal Antibody (7F6G6)**
**Seq50'**	**Cy7**	**AF488**	**Cy3**	**JNK Monoclonal Antibody (1A12E1)**

Elution Process Decoding

**cDNA**	**cDNA-dye cocktail 1**	**cDNA-dye cocktail 2**	**cDNA - dye cocktail 3**	**Round 1 (25)**	**Round 2 (25)**	**Round 3 (25)**	**Round 4 (25)**	**Round 5 (25)**
	**Cycle1**	**Cycle2**	**Cycle3**					
**Seq1'**	**AF488**	**AF488**	**AF488**		**Ephrin B1/B2**		**JAK3 Polyclon**	

					**Polyclonal Antibody**		**al Antibody**	
**Seq2'**	**AF488**	**AF488**	**Cy3**		**HMGCS1 Polyclonal Antibody**		**Protein APC Polyclonal Antibody**	
**Seq3'**	**AF488**	**AF488**	**Cy5**		**Phospho-Amyloid Precursor Protein (Thr743, Thr668) Antibody**		**Phospho-EGFR (Ser1047) Polyclonal Antibody**	
**Seq4'**	**AF488**	**AF488**	**Cy7**		**ASAH1 Antibody**		**GNAI3 Polyclonal Antibody**	
**Seq5'**	**AF488**	**Cy3**	**AF488**		**Phospho-IP3 Receptor 1 (Ser1588, Ser1598) Antibody**		**Human/Mouse/Rat Phospho-Akt (S473) Pan**	
**Seq6'**	**AF488**	**Cy3**	**Cy3**		**Phospho-Calmodulin 1/2/3 (Thr79, Ser81) Antibody**		**Human/Mouse Phospho-STAT1 (Y701)**	
**Seq7'**	**AF488**	**Cy3**	**Cy5**		**Phospho-SREBP1 (Ser439) Polyclonal**		**Purified anti-HSP60 Antibody**	

					Antibody			
Seq8'	AF488	Cy3	Cy7		COX2 Polyclonal Antibody		Human/ Mouse TOR	
Seq9'	AF488	Cy5	AF488		TrkA Polyclonal Antibody		Mouse Notch-1 Antibody	
Seq10'	AF488	Cy5	Cy3		Dynamin 1 Polyclonal Antibody		Mouse Notch-2 Antibody	
Seq11'	AF488	Cy5	Cy5		CHRFAM7A Polyclonal Antibody		Mouse Notch-3 Antibody	
Seq12'	AF488	Cy5	Cy7		HMGCR Polyclonal Antibody		Human/ Mouse/ Rat Phospho-GSK-3 alpha / beta (S21/S9) Antibody	
Seq13'	AF488	Cy7	AF488		Human/ Mouse/ Rat GSK-3 alpha / beta Antibody		INSR Polyclonal Antibody	
Seq14'	AF488	Cy7	Cy3		Mu-Calpain Polyclonal		Human/ Mouse/ Rat PDK-1	

					Antibody		Antibody		
Seq15'	Cy3	Cy7	Cy5		TrkB Polyclonal Antibody		Human/ Mouse/ Rat PTEN Antibody		
Seq16'	Cy3	Cy7	Cy7		FOXO1 Monoclonal Antibody		Human/ Mouse/ Rat eIF4E Antibody		
Seq17'	Cy3	AF488	AF488		APOE Recombinant Antibody		Human/ Mouse/ Rat Src Antibody		
Seq18'	Cy3	AF488	Cy3		PAK1 Polyclonal Antibody		Human/ Mouse/ Rat TSC1 Antibody		
Seq19'	Cy3	AF488	Cy5		TREM2 Recombinant Antibody		Human/ Mouse TSC2 Antibody		
Seq20'	Cy3	AF488	Cy7		Phospho-CREB/ATF1 (Ser133, Ser63) Monoclonal Antibody		Human/ Mouse/ Rat p70 S6 Kinase Antibody		
Seq21'	Cy3	Cy3	AF488		LEF1 Polyclonal Antibody		Human/ Mouse/ Rat Rheb Antibody		

**Seq22'**	**Cy3**	**Cy3**	**Cy3**		**Phospho-JNK1/JNK2 (Thr183, Tyr185) Monoclonal Antibody**		**Human/ Mouse/ Rat GRB2 Antibody**	
**Seq23'**	**Cy3**	**Cy3**	**Cy5**		**PDCD4 Recombinant Rabbit Monoclonal Antibody**		**Human/ Mouse STAT5a Antibody**	
**Seq24'**	**Cy3**	**Cy3**	**Cy7**		**EIF2S1 Monoclonal Antibody (EIF2-alpha)**		**Human/ Mouse STAT5b Antibody**	
**Seq25'**	**Cy3**	**Cy5**	**AF488**		**K-Ras Monoclonal Antibody (9.13)**		**Human/ Mouse/ Rat STAT6 aa 627-838 Antibody**	
**Seq26'**	**Cy3**	**Cy5**	**Cy3**	**anti-Tubulin β3**		**Arp2 Recombinant Rabbit Monoclonal Antibody (5H2L7)**		**Human/ Mouse/ Rat Jak1 Antibody**
**Seq27'**	**Cy5**	**Cy5**	**Cy5**	**anti-MAP2 Antibody**		**Phospho-BRAF (Ser729) Polyclonal**		**Human/ Mouse Beclin 1 Antibody**

						Antibody		
Seq28'	Cy5	Cy5	Cy7	anti-NSE Antibody		CK1 gamma-1 Polyclonal Antibody		Human/Mouse/Rat p27/Kip1 Antibody
Seq29'	Cy5	Cy7	AF488	anti-p62 (SQSTM1) Antibody		Tau Monoclonal Antibody		Human/Mouse CDK4 Antibody
Seq30'	Cy5	Cy7	Cy3	anti-MAGUK (pan reactive) Antibody		NLK Polyclonal Antibody		Human/Mouse/Rat CDK2 Antibody
Seq31'	Cy5	Cy7	Cy5	anti-Neuroligin-1 Antibody		Purified anti-MEK1 Phospho (Ser298) Antibody		Human/Mouse Cyclin D1/D2 Cross-reactive Antibody
Seq32'	Cy5	Cy7	Cy7	anti-VGlut1 Antibody		Phospho-p70 S6 Kinase (Ser424) Polyclonal Antibody		Human/Mouse/Rat MDM2/HDM2 Antibody
Seq33'	Cy5	AF488	AF488	Dopamine Transporter Polyclonal Antibody		Phospho-beta Catenin (Thr41, Ser45) Polyclonal Antibody		Human/Mouse Active Caspase-3 Antibody
Seq34'	Cy5	AF488	Cy3	anti-UFD1		Phospho-BAD		Human/Mouse

						(Ser155) Polyclonal Antibody		Bad aa 1-21 Antibody
**Seq35'**	**Cy5**	**AF488**	**Cy5**	**anti-mouse/human CD11b**		**GNAQ Polyclonal Antibody**		**Human/ Mouse/ Rat BAK Antibody**
**Seq36'**	**Cy5**	**AF488**	**Cy7**	**anti-mouse CD16/32**		**Phospho-TAK1 (Thr187) Polyclonal Antibody**		**Human/ Mouse PAK3 Antibody**
**Seq37'**	**Cy5**	**Cy3**	**AF488**	**anti-mouse CD40**		**Phospho-SHP2 (Tyr62) Polyclonal Antibody**		**Human/ Mouse/ Rat p53 Antibody**
**Seq38'**	**Cy5**	**Cy3**	**Cy3**	**anti-mouse CD45**		**MRLC2 Polyclonal Antibody**		**Human/ Mouse c-Myc Antibody**
**Seq39'**	**Cy7**	**Cy3**	**Cy5**	**anti-mouse CD68**		**Caspase 9 (Cleaved Asp330) Polyclonal Antibody**		**Human/ Mouse IkB-alpha Antibody**
**Seq40'**	**Cy7**	**Cy3**	**Cy7**	**anti-mouse CD86**		**Phospho-PKA alpha (Ser338) Polyclonal Antibody**		**Human/ Mouse/ Rat RelA/NF kappa B p65 Antibody**
**Seq41'**	**Cy7**	**Cy5**	**AF488**	**anti-mouse I-**		**BID Polyclon**		**Human/ Mouse**

				**A/I-E Antibody**		**al Antibody**		**cIAP-2/HIAP-1 Antibody**
**Seq42'**	**Cy7**	**Cy5**	**Cy3**	**anti-mouse CD206 (MMR)**		**AXIN1 Polyclonal Antibody**		**Human/ Mouse/ Rat/Chicken Oligodendrocyte Marker O4 Antibody**
**Seq43'**	**Cy7**	**Cy5**	**Cy5**	**anti-mouse CD301a (MGL1)**		**GLUT4 Monoclonal Antibody (3G10A3)**		**RHOA Polyclonal Antibody**
**Seq44'**	**Cy7**	**Cy5**	**Cy7**	**anti-mouse CD163**		**Purified anti-Hsp90α/β Antibody**		**TNFR1 Monoclonal Antibody (2A6E3)**
**Seq45'**	**Cy7**	**Cy7**	**AF488**	**anti-mouse/human CD15 (SSEA-1)**		**Lamin A/C Polyclonal Antibody**		**Rac1 Monoclonal Antibody (4A4B11)**
**Seq46'**	**Cy7**	**Cy7**	**Cy3**	**anti-mouse CD115 (CSF-1R)**		**CDK5 Polyclonal Antibody**		**NOL3 Polyclonal Antibody**
**Seq47'**	**Cy7**	**Cy7**	**Cy5**	**anti-mouse CD64 (FcγRI)**		**ROCK1 Polyclonal Antibody**		**PI3K p85 alpha Monoclonal Antibody**

**Seq48'**	**Cy7**	**Cy7**	**Cy7**	**anti-mouse CD209a (DC-SIGN) Antibody**		**ERAB Polyclonal Antibody**		**4EBP1 Monoclonal Antibody (2C3F3)**
**Seq49'**	**Cy7**	**AF488**	**AF488**	**anti-SNAP-25 Antibody**		**IDE Polyclonal Antibody**		**SIRT1 Monoclonal Antibody (7F6G6)**
**Seq50'**	**Cy7**	**AF488**	**Cy3**	**anti-Myelin Basic Protein Antibody**		**PKC alpha Polyclonal Antibody**		**JNK Monoclonal Antibody (1A12E1)**

Microbeads used in Spatial MIST

The following images show the beads that were used in the experiments for this work. Beads were conjugated with specific oligonucleotide sequences then used to make MIST arrays.

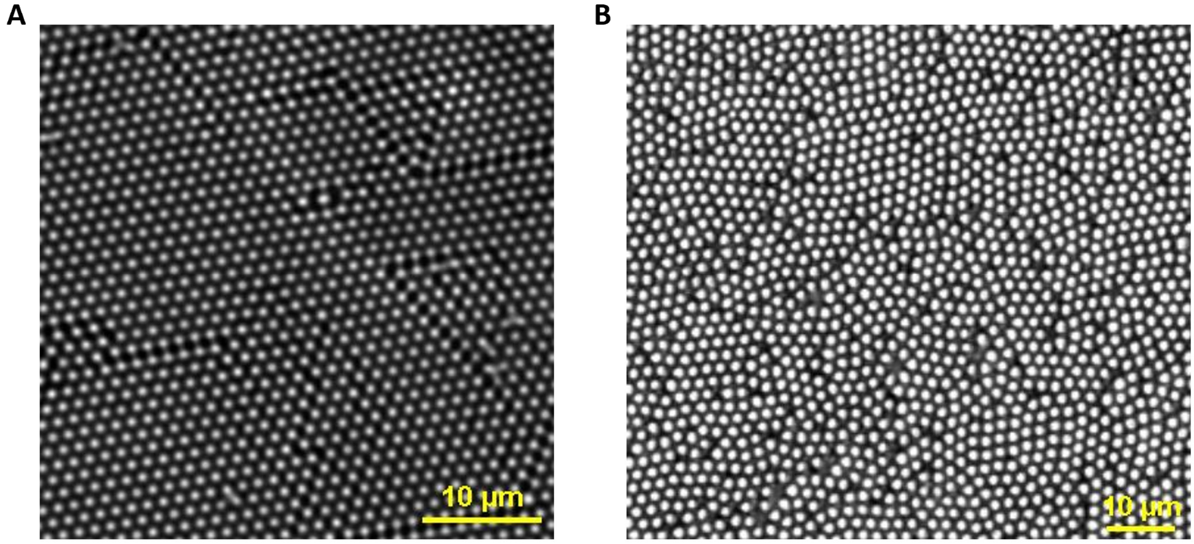

Figure S1. *Microbeads used to make MIST arrays. (**A**) Close up view of 1.4 µm carboxyl latex beads taken with a 40x objective. (**B**) Close up view of 2 µm amine latex beads taken with 20x objective.*

Varying Concentrations of 8-arm PEG

In order to find the optimum concentration of 8-arm PEG for our experiment, we tested the signal intensity and distribution of several concentrations of PEG. We found that 30% PEG enabled sharpest images without weakening signal too much.

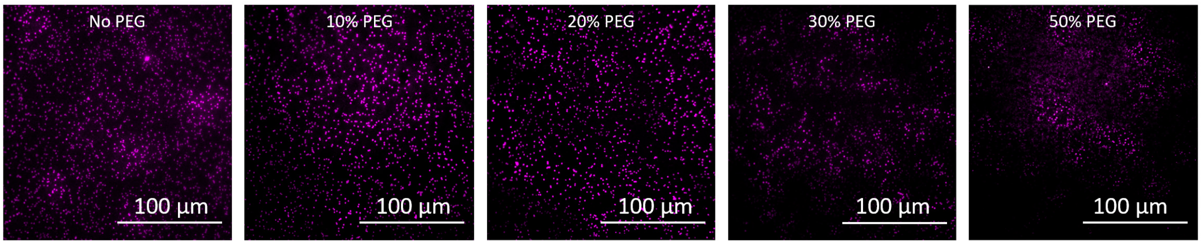

Figure S2. *Effects of varying Concentrations of 8-arm PEG added before UV cleavage on Spatial MIST array from cortical region of mouse brain. FOX3 protein is being detected in all images. The left most image is control. PEG concentration is increasing from left to right.*
